# Application of the Nagoya Protocol to veterinary pathogens: concerns for the control of foot-and-mouth disease

**DOI:** 10.3389/fvets.2023.1271434

**Published:** 2023-11-22

**Authors:** Jacquelyn Horsington, Elke Abbeloos, Labib Bakkali Kassimi, Kingkarn Boonsuya Seeyo, Alejandra V. Capozzo, Eunice Chepkwony, Phaedra Eblé, Sabrina Galdo-Novo, Daniel Gizaw, Lizelle Gouverneur, Santina Grazioli, Livio Heath, Pascal Hudelet, Joseph M. K. Hyera, Martin Ilott, Alasdair King, David J. Lefebvre, David Mackay, Samia Metwally, Frank N. Mwiine, Charles K. Nfon, Min-Kyung Park, Edviges Maristela Pituco, Fabrizio Rosso, Francisco Simon, Hussaini G. Ularamu, Paul Vermeij, Wilna Vosloo, Donald P. King

**Affiliations:** ^1^European Commission for the Control of Foot-and-Mouth Disease (EuFMD), Rome, Italy; ^2^Boehringer Ingelheim Animal Health, Lyon, France; ^3^ WOAH/FAO FMD Reference Laboratory Network; ^4^Animal Health Laboratory, UMR1161 Virology, INRAE, ANSES, ENVA, Paris-Est University, Maisons-Alfort, France; ^5^Regional Reference Laboratory for FMD in Southeast Asia, Pakchong, Thailand; ^6^Global Foot-and-Mouth Disease Research Alliance (GFRA); ^7^Foot and Mouth Disease National Laboratory, Embakasi, Directorate of Veterinary Services, State Department of Livestock, Nairobi, Kenya; ^8^Wageningen Bioveterinary Research (WBVR), Lelystad, Netherlands; ^9^Servicio Nacional de Sanidad y Calidad Agroalimentaria (SENASA), Buenos Aires, Argentina; ^10^Animal Health Institute (AHI), Sebeta, Ethiopia; ^11^FAO World Reference Laboratory for FMD, The Pirbright Institute, Pirbright, United Kingdom; ^12^Istituto Zooprofilattico Sperimentale della Lombardia e dell'Emilia-Romagna, Brescia, Italy; ^13^Transboundary Animal Disease Laboratory, Onderstepoort Veterinary Institute, Agricultural Research Council, Onderstepoort, South Africa; ^14^WOAH Reference Laboratory for FMD, Botswana Vaccine Institute, Lejara, Gaborone, Botswana; ^15^MSD Animal Health, Boxmeer, Netherlands; ^16^Sciensano, Scientific Direction of Infectious Diseases in Animals, Service for Exotic and Vector-borne Diseases, Brussels, Belgium; ^17^Food and Agriculture Organization of the United Nations (FAO), Rome, Italy; ^18^College of Veterinary Medicine, Animal Resources, and Biosecurity, Makerere University, Kampala, Uganda; ^19^Canadian Food Inspection Agency, National Centre for Foreign Animal Disease, Winnipeg, MB, Canada; ^20^World Organisation for Animal Health (WOAH), Paris, France; ^21^Pan American Health Organization, Regional Office for the Americas of the World Health Organization, Rio de Janeiro, Brazil; ^22^Biogenesis Bago, Buenos Aires, Argentina; ^23^Viral Research Division, National Veterinary Research Institute, Vom, Nigeria; ^24^Australian Centre for Disease Preparedness, Commonwealth Scientific and Industrial Research Organisation (CSIRO) Health and Biosecurity, Geelong, VIC, Australia

**Keywords:** foot-and-mouth disease, Nagoya Protocol, access and benefit sharing, vaccine security, utilization

## Abstract

The Nagoya Protocol is an international agreement adopted in 2010 (and entered into force in 2014) which governs access to genetic resources and the fair and equitable sharing of benefits from their utilisation. The agreement aims to prevent misappropriation of genetic resources and, through benefit sharing, create incentives for the conservation and sustainable use of biological diversity. While the equitable sharing of the benefits arising from the utilisation of genetic resources is a widely accepted concept, the way in which the provisions of the Nagoya Protocol are currently being implemented through national access and benefit-sharing legislation places significant logistical challenges on the control of transboundary livestock diseases such as foot-and-mouth disease (FMD). Delays to access FMD virus isolates from the field disrupt the production of new FMD vaccines and other tailored tools for research, surveillance and outbreak control. These concerns were raised within the FMD Reference Laboratory Network and were explored at a recent multistakeholder meeting hosted by the European Commission for the Control of FMD. The aim of this paper is to promote wider awareness of the Nagoya Protocol, and to highlight its impacts on the regular exchange and utilisation of biological materials collected from clinical cases which underpin FMD research activities, and work to develop new epidemiologically relevant vaccines and other diagnostic tools to control the disease.

## 1 Introduction

### 1.1 History and motivation behind the establishment of an international treaty on genetic resources

The Global North benefiting from the appropriation of resources from the Global South, often without fair recompense, is a common theme in history, and the exploitation of natural biodiversity and resources present in developing nations by developed countries is a topic that is widely discussed (1–3). In order to address some of this inequity, the international community recognised that all countries should have sovereign rights over their own biological resources, and advocated for regulation of bioprospecting activities conducted in biodiversity-rich countries by users based in other countries (4, 5). The creation of a global framework for access and benefit-sharing (ABS) of genetic resources ensures that the users of these resources share the benefits (financial and other) generated through their use, and for the provider countries to then reinvest those benefits into conservation of biodiversity. These concepts were formerly recognised by the Convention on Biological Diversity (CBD), which was adopted in May 1992 with near universal ratification. The three overarching objectives of the CBD are (i) the conservation of biological diversity, (ii) the sustainable use of the components of biological diversity, and (iii) the fair and equitable sharing of the benefits arising out of the utilisation of genetic resources (6).

**Figure 1:**
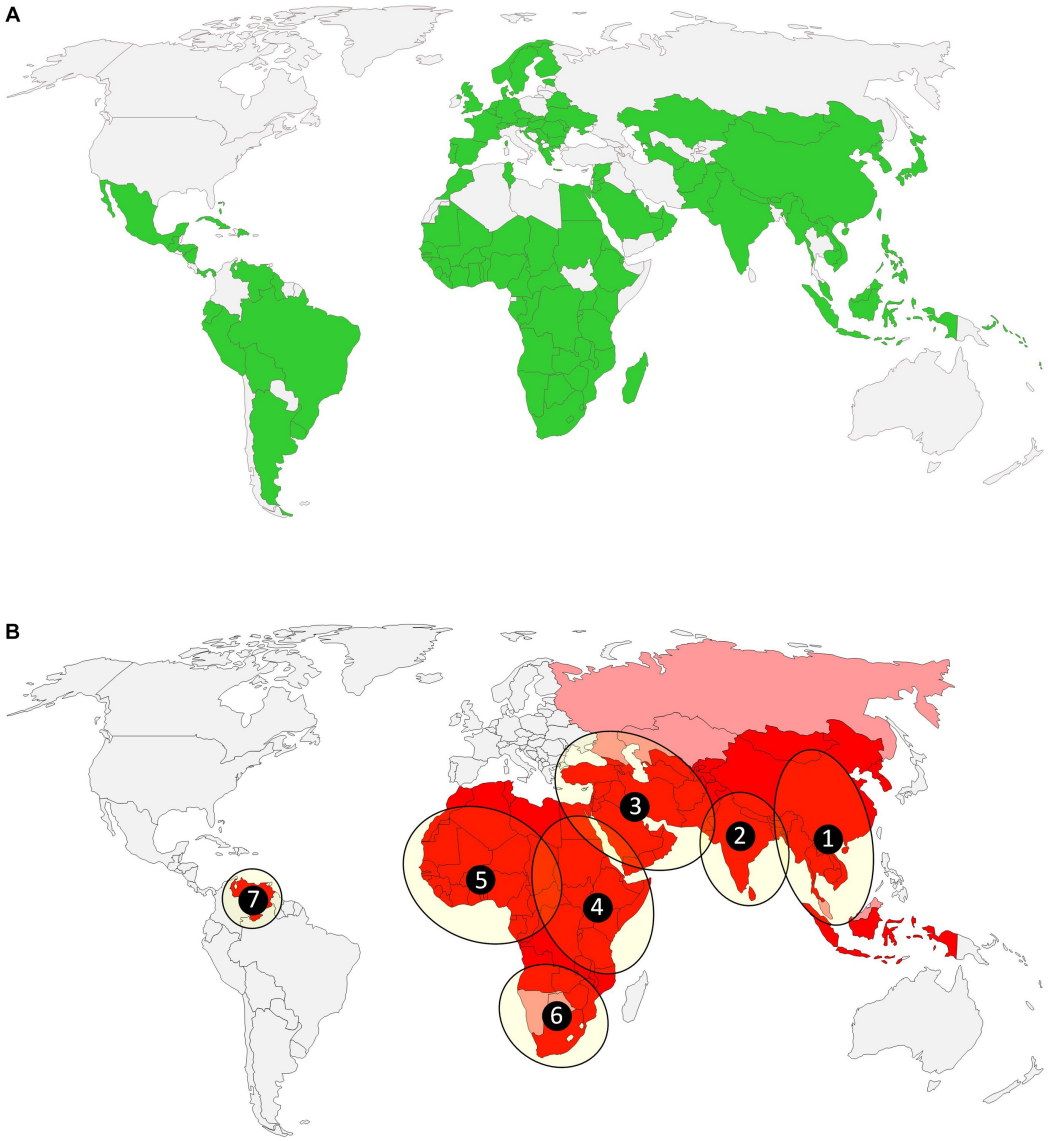
Countries that are parties to the Nagoya Protocol **(A)** and countries where FMD is present within seven endemic virus pools (1–7) located in Asia, Africa and South America **(B)**. Grey colour defines countries that maintain an FMD-free status (with or without vaccination), dark red represents countries without any FMD-free status, while lighter red denotes countries with at least one FMD-free zone (as defined by WOAH in March 2023).

Building on this framework, the Nagoya Protocol was adopted at the tenth meeting of the Conference of the Parties to the CBD (COP10), held in Nagoya, Japan in 2010 (7) and came into force in 2014. The Nagoya Protocol is a supplementary agreement to the CBD and aims to ensure international equity with regard to “sharing of the benefits arising from the utilisation of genetic resources, including by appropriate access to genetic resources and by appropriate transfer of relevant technologies, taking into account all rights over those resources and to technologies, and by appropriate funding, thereby contributing to the conservation of biological diversity and the sustainable use of its components” (8). At the time of writing (June 2023), there are 139 countries that have either ratified, accepted, approved, or acceded to this agreement (Figure 1A). The principle of this legally binding framework is that ABS is agreed upon on a bilateral basis between users and the provider country through the negotiation of prior informed consent (PIC) and mutually agreed terms (MAT). The PIC must ensure that the party providing consent fully understands how the user intends to make use of the genetic resource, and the MAT acts as an agreement as to what the expected benefit will be (monetary or otherwise) and how this will be shared. Access and benefit-sharing obligations are regulated at a national level, that is, regulated under national laws of the country providing the genetic resource, which may be the country of origin of such resources or a Party that has acquired the genetic resources in accordance with the Convention.

### 1.2 Scope of the Nagoya Protocol

A key principle recognised by the CBD and further operationalised under the Nagoya Protocol is that countries have sovereign rights over their natural resources and, thus, the authority to determine access to genetic resources lies with national governments and is subject to the national legislation of the country in which they are found, thereby giving countries the ability to determine, control, and monitor the use of any such genetic resources accessed within their territory. The scope of the Nagoya Protocol encompasses the genetic material of plants, animals, and microorganisms, although it does not apply to human genetic material (8). Access and benefit-sharing legislation can also apply to derivatives and biochemical compounds extracted from these genetic resources (4). The terms negotiated for the sharing of benefits arising from the utilisation of such resources can cover a range of monetary and non-monetary benefits, including royalties and licence fees, data management, dissemination of research and development results, collaboration in scientific programmes, technology transfer and capacity building (8, 9). Importantly, this may be interpreted to include greater equity in access to medical advances, such as vaccines, which historically has been lacking. Utilisation of genetic resources means to conduct research and development on the genetic and/or biochemical composition of genetic resources, including through the application of biotechnology (4). Therefore, purely descriptive research, as well as activities where the genetic resource is used as a tool for characterisation, are considered by many national ABS measures as being outside of scope, e.g., taxonomic identification, using the genetic resource as reference material in tests or for diagnostic purposes (Table 1). Since countries have sovereign rights over genetic resources within their jurisdiction, the actual scope of ABS measures is defined by national law. The national laws of some countries may provide for a broader scope than the Nagoya Protocol.

**Table 1:** Nagoya Protocol: impacts* on specific examples of FMD surveillance, academic research and commercial activities.

Uses of material that is probably in scope (depending on the applicable national regulations) Any genetic resource that is the subject of utilisation, i.e., research and development on the genetic or biochemical composition of the genetic resource, e.g., material that is the subject of research aimed at discovering or examining specific characteristics of the material (even if it is academic research), or to develop a product. This includes:	Material that may be out of scope, depending on the national regulations of the provider and user countries Any genetic resource that is not the subject of utilisation, i.e., no research and/or development is taking place on the genetic or biochemical composition of the genetic resource. This may include:
A field strain of FMDV used to develop a vaccine seed stock	Material used for taxonomy or identification of a resource. For instance, confirmatory testing or sequencing of samples received by a FMD reference laboratory.
An FMD virus being developed to become a component of a therapeutic agent	Antigenic characterisation of field strains (vaccine matching)
An FMD virus being developed to become a component of a diagnostic test	FMD viruses used as challenge strains in animal trials
An FMD virus being developed to become a research tool or to generate new knowledge	Materials used as a positive or negative controls for in-house tests
Production of recombinant FMDV proteins for research or commercial purposes that utilise specific genetic information derived by the user from field viruses (this depends on the legal status of DSI in the relevant national ABS regulation)	Samples used as components in a proficiency testing panel
Contract research that includes any of the above examples – responsibility for compliance should be made clear between the customer and the service provider	Long-term archiving of materials

*This table provides an interpretation of the ways in which FMD materials may be used. This should not be considered as a definitive interpretation of the CBD, the Nagoya Protocol, or national ABS requirements as it remains the responsibility of national competent authorities to interpret these requirements as they see fit.

Legislation on the sourcing and use of genetic resources includes administrative procedures and enforcement policies that vary from country to country. In its simplest form, a foreign researcher/company contacts the provider country’s national focal point (NFP; an administrative contact person) to obtain information on applicable procedures for obtaining PIC and establishing MAT, including benefit-sharing. With this information, the researcher/company will then contact the country’s national competent authority (NCA) to initiate negotiations on PIC and MAT. The NCA may be the ministry of the environment, the ministry of health, indigenous issues, interior, or some other department, and in some countries multiple ministries may claim jurisdiction over the resource (10). The PIC and/or MAT finally agreed should detail the conditions of access, use and further sharing of the genetic resource and the sharing of benefits arising from its utilisation.

While pathogens clearly represent genetic resources as defined in the Nagoya Protocol, provisions to recognise specific and unique characteristics of pathogens, and clarification or further guidance on whether or not parties should include them within the scope of their domestic ABS laws, are lacking. Of the 139 countries Party to the Nagoya Protocol, 77 have ABS rules in place that cover “microorganisms,” though not referring specifically to “pathogens” (5). The inclusion of pathogens in the scope of the ABS framework, but the uncertainty about how to manage this, has sparked debate on the potentially negative impacts of the Nagoya Protocol and related ABS laws on human and animal health.

Article 8(b) of the Nagoya Protocol calls on parties to “pay due regard to cases of present or imminent emergencies that threaten or damage human, animal and/or plant health, as determined nationally or internationally.” In these situations, parties may take into consideration the need for expeditious access to genetic resources and fair and equitable sharing of the benefits (8, 11). In addition, Article 8(c) requests consideration of the importance of genetic resources for food and agriculture and their special role for food security (8). However, the Protocol does not provide any guidance as to how these “special considerations” should be implemented in practice, with some countries adopting applying specialised provisions to pathogens and others yet to address the issue.

Whether or not digital sequence information (DSI) on genetic resources (such as sequences from pathogens) is to be regarded as a genetic resource, as defined by the CBD, is controversial. However, various countries have already included DSI in their ABS legislation. This can impact access to and exchange of DSI, for example, between international reference laboratories or in sequence databases. At the most recent COP15 of the CBD, held in December 2022, discussions on DSI led to an agreement by Parties to establish a multilateral mechanism for benefit sharing from the use of DSI on genetic resources, including a global fund (12, 13). The specific details of this mechanism are still to be determined.

### 1.3 Legal complexity of the Nagoya Protocol and its national operationalisation

The Nagoya Protocol does not harmonise ABS measures at the global level. Instead, it defines a set of principles and establishes certain mechanisms that require implementation by its Parties. The actual ABS obligations for users of genetic resources are defined by national legislative, administrative and policy measures. This results in a high level of heterogeneity in definitions, obligations and procedures among provider countries [Table 2; (14, 15)]. In addition, establishing effective national legislation can be hindered by lack of budget or technical expertise, lack of strong government structures or political support, as well as by conflict over ownership of the genetic resources of interest (16, 17). The increased administrative burden and multi-layer decision-making process causes complications and the potential for confusion for both providers and users.

**Table 2:** The impact of interpretation* of the Nagoya Protocol into national ABS legislation on the exchange of FMD materials.

Material that *is in principle* out of scope of the Nagoya Protocol…	…but sometimes *may* result in ABS obligations
Material that is not a genetic resource, i.e., does not contain functional units of heredity	Derivatives of genetic resources, i.e., naturally occurring biochemical compounds resulting from the genetic expression or metabolism of the genetic resource, such as proteins or lipids.
Material obtained from a country that, at the time of access, was not a Party to the Protocol	Some countries which are not a Party to the Nagoya Protocol have national (or regional) ABS laws (e.g., Australia) and certain obligations may arise in relation to materials obtained after 1993 when the CBD entered into force
Material obtained before 12 October 2014	Some countries have had national ABS laws in place before 2014 (e.g., Brazil). Certain countries provide for a *de facto* retroactive effect of their national ABS laws and certain obligations may arise in relation to materials obtained after 1993 when the CBD entered into force
Human genetic resources	
Genetic resources covered by a Specialised International ABS Instrument, for instance the International Treaty on Plant Genetic Resources for Food and Agriculture.	If used outside the parameters of the Specialised International ABS Instrument (e.g., plant genetic resources not used for food or agricultural purposes)
Material traded and used as commodities	If a material originally traded as a commodity becomes the subject of research and development. Certain countries may also cover the trade of material as commodities, for example as “biotrade.”
Synthetic material	If the material is synthesised by using digital sequence information on genetic resources (note that the treatment of digital sequence information is still under international discussion, but in some countries already covered by national ABS laws)
Material unintentionally accessed, e.g., a tissue sample that contains the pathogen of interest, as well as other pathogens and genetic material (e.g., from the animal sampled) that will not be utilised	If material unintentionally accessed becomes the subject of research and development.

*The Nagoya Protocol is an international agreement that establishes a regime within the context of the CBD that promotes and safeguards the fair and equitable sharing of benefits arising from utilisation of genetic resources. The CBD and the Nagoya Protocol are implemented by means of national ABS legislation introduced by the signatories to the Convention and the Protocol. Each country may interpret the requirements of the Convention and the Protocol differently and may choose to extend national ABS provisions beyond those specified in either agreement. The complex interaction of these three levels of requirements leads to different controls for the same type of material between different countries making a single interpretation impossible. This table provides a broad, non-exhaustive summary of the way in which the provisions are generally interpreted by laboratories with respect to what FMD materials may potentially be in or out of scope of national ABS obligations under the Nagoya Protocol. This should not be considered as a definitive interpretation of the CBD, the Nagoya Protocol, or national ABS requirements as it remains the responsibility of national competent authorities to interpret these requirements as they see fit.

Non-compliance with (national) ABS laws potentially has severe consequences, including fines and criminal sentences (18, 19). This applies whether a country is a party to the Nagoya Protocol or not. If a genetic resource originates from a country party to the CBD that has not ratified the Nagoya Protocol, but has implemented ABS legislation (examples include: Iran, Thailand, Australia) compliance with that Party’s measures is equally required. In addition, if a genetic resource originates from a country that has ratified the Nagoya Protocol (Figure 1A), additional compliance measures in the country of utilisation might be required. For example, in the EU prior to the release of a product onto the market, any product or technology based on a genetic resource will be subject to specific due diligence obligations. The legal uncertainty, especially in this complex heterogenous environment, creates legal risks for companies and institutions.

There is an urgent need to clarify the ABS framework and related processes for both users and providers and for a greater science-policy dialogue both within and among countries to ensure a better understanding and more effective implementation of the Nagoya Protocol and related ABS measures. At COP15, parties to the CBD also adopted the Kunming-Montreal Global Biodiversity Framework, comprising four global goals contributing to the three objectives of the CBD. The third goal specifically refers to sharing of benefits from the use of genetic resources. The framework seeks to facilitate enhanced synergy between the CBD, its Protocols and other relevant multilateral agreements, and international organisations and processes. It notes the importance of One Health and food security and encourages taking effective legal, policy, administrative, and capacity-building measures at all levels, as appropriate, to ensure the fair and equitable sharing of benefits that arise from the utilisation of genetic resources and from DSI (12, 13). Whether or not this framework will promote better cooperation, understanding, and solutions for ABS in relation to pathogens remains to be seen, but the focus on a comprehensive approach to ensure effective measures at all levels should be supported.

### 1.4 Viral sovereignty

Viral sovereignty is the concept that countries have sovereign rights over viruses located within their jurisdiction and, therefore, may determine access to these viruses - a key concept that flows from the CBD and the application of the Nagoya Protocol to pathogen sharing. This came into focus in the mid-2000s when Indonesia was reluctant to share H5N1 influenza viruses isolated in the country with the WHO’s Global Influenza Surveillance and Response System (GISRS) until agreements granting it access to antivirals and vaccines were formulated. Indonesia challenged the expectation that virus samples should be shared with WHO without consideration of fair access to any vaccines resulting from those samples, highlighting the exploitation of developing provider countries. This was the first time that the CBD was explicitly invoked in a case concering access to human pathogens (10, 20). However, this concept of viral sovereignty, combined with political complexities, has been shown to have negative impacts on public health situations. In 2016, access to Zika virus samples and data from the outbreak in Brazil was inhibited largely due to the Brazilian ABS laws affecting material transfer (10, 21). However, negotiation of access terms became redundant when the virus spread to Puerto Rico facilitating easy access by researchers at the US Centres for Disease Control and Prevention (20, 22). Similarly, controversy regarding sovereignty claims by the government of Saudi Arabia over MERS-CoV, and the complex legal situation that ensued, prevented sample sharing and impeded research on antivirals and vaccines against the virus (5, 10, 22). As phrased by Rouke, “Viruses are unequivocally *genetic resources* within the remit of the CBD. But this does not necessarily mean that they should be.” (22) Thus, implementation of the Nagoya Protocol, and any national ABS laws, need to balance the legitimate expectations of the provider country with the need to adopt a simplified and equitable process that does not impede the development of tools that are required for global health and food security.

### 1.5 The impacts of the Nagoya Protocol on FMD research and control

Foot-and-mouth disease (FMD) is an economically important disease of livestock and is present in Africa, Asia, and parts of South America (Figure 1B). The disease is characterised by fever, lameness and the appearance of vesicular lesions on the mouth, tongue, nose, feet and teats, and can be associated with reduced milk yield and abortions. In endemic countries, FMD causes major economic losses to the agriculture sector, while in countries that are free from the disease it poses the continuous threat of devastating outbreaks with impacts far beyond the agriculture sector (23). FMD virus (FMDV) is a small, positive-sense, single-stranded RNA virus in the genus *Aphthovirus*, family *Picornaviridae*. It has a very dynamic and complex epidemiology, with six actively circulating serotypes (a seventh serotype, type C, is most likely extinct (24)). There is no cross-protection between serotypes and even within serotypes cross-protection can be limited due to the presence of distinct antigenic variants. Globally, FMD viruses are grouped into seven geographically distinct virus pools. Each pool contains viruses of multiple serotypes, with varying numbers of specific topotypes within each serotype. The virus evolves rapidly to continuously generate new lineages which can escape virus neutralisation induced by existing vaccine strains potentially causing devastating disease outbreaks. Vaccination in endemic countries is a control option that is widely used to protect animals and ensure livestock production sustainability and thus food supply (25), while FMD-free countries rely on strategic stocks (“banks”) of vaccine antigens that can be formulated quickly in response to FMDV incursions. These factors mean that there is a constant need to (i) monitor the antigenic diversity of field viruses (necessitating access to viruses by international reference laboratories) and (ii) ensure that new vaccine strains can be produced that are tailored to antigenically distinct lineages. While manufacturers of human vaccines can sometimes take advantage of sourcing a pathogen from the “returning traveller,” this is by default not possible for FMD vaccine manufacturers, since trade rules established by the World Organisation for Animal Health (WOAH) prevent any traffic of FMD susceptible animals or untreated products of animal origin from endemic zones.

## 2 Specific impacts of the Nagoya Protocol on key FMD stakeholders

### 2.1 Diagnostic/reference laboratories

**Figure 2:**
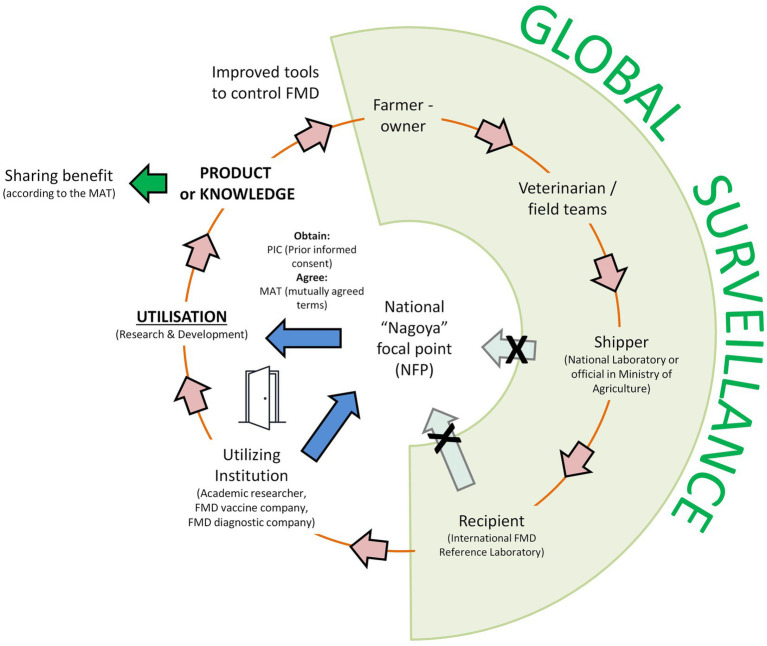
Feedback loop (pink arrows) for the *utilisation* of FMD resources highlighting the main actors in this process. NB: the shipment of samples to international FMD reference Laboratories does not typically involve the National NFP and their engagement in the process usually only occurs prior to utilisation of the samples once samples have been tested and characterised. Any measures to simplify the process to access FMD samples from endemic countries need to provide legal certainty and thereby ensure that global surveillance activities relating to FMD are not unintentionally impeded.

Global surveillance of FMD coordinated by the WOAH/FAO FMD Reference Laboratory Network^1^ involves the characterisation of FMDV positive samples collected from field cases in endemic countries and outbreaks (Figure 2). During the past 5 years (2018–2022), Network laboratories have tested >10,000 samples from FMD cases collected in 64 countries. While these immediate diagnostic activities are widely interpreted as falling outside of the scope of “utilisation” as defined by the Nagoya Protocol, the long-term storage, distribution, and further use of these diagnostic samples and/or isolated strains often might become restricted by the ABS framework (Table 1). In addition to the downstream use of these materials for basic and applied research activities, FMD reference laboratories often play an important role to supply field isolates to commercial vaccine companies so that they can develop new master seed strains to cover emerging viral strains.

It is important to note that scientists within international FMD reference laboratories and their partners in FMD endemic countries often lack specific expertise on the Nagoya Protocol or ABS and currently do not have the knowledge or resources to make contact or prepare agreements with the NFPs or NCAs in their own country. Furthermore, since confirmatory diagnostic testing and strain characterisation are widely accepted as falling outside of the scope of the Nagoya Protocol, the NFPs do not normally have sight of these activities, particularly as samples are sent to international reference laboratories at the discretion of local laboratories (Figure 2). Therefore, whilst laboratory staff are experienced in the despatch and receipt of diagnostic samples, they are often uncertain about their potential liability in terms of ABS obligations with respect to the downstream utilisation of these materials by third parties. This situation is complicated by the collaborative relationships that are often established to share samples, where co-authorship of scientific papers is usually considered to be the most appropriate way to equitably share the benefits of the work associated with the use of field materials.

### 2.2 Vaccine manufacturers

The Nagoya Protocol can have a significant impact on the response time of vaccine manufacturers to provide vaccines. In endemic areas, FMDV may evolve rapidly requiring vaccine manufacturers to update FMD vaccines periodically to match the changing epidemiology of the virus in the field. The sourcing of pathogens for the development of a vaccine is considered “utilisation of genetic resources” and as such falls within the scope of the Nagoya Protocol (Figure 2). Consequently, unless the relevant country decided not to regulate ABS, there is an obligation to obtain PIC and to establish MAT for access to and use of FMDV field strains. Vaccine manufacturers are frequently constrained by commercial confidentiality from making public the basis on which they obtain the strains of FMDV used in their vaccines. Nevertheless, at least one manufacturer has highlighted that to their knowledge despite sustained and repeated attempts, successful conclusion of this process has not been achieved by any pharmaceutical company with respect to FMD viruses in situations where countries have started to implement ABS provisions at national level (26). It should also be noted that the requirement to establish separate agreements with individual countries, and often the legal requirement to source viruses from a local laboratory rather than the World Reference Laboratory, may constrain the ability of the companies to screen a wide range of viruses to quickly select those with the best characteristics for use as potential vaccine candidates. In situations where there is an urgent need for a new vaccine strain to control newly emerged field strains, any delay to new vaccine development has a direct impact on people’s livelihoods, food security and risk for incursion in FMD free areas.

While manufacturers respect the principle of sovereignty of genetic resources and agree with the principle of fair benefit sharing, experience has shown that there may be unrealistic expectations among provider countries about the scale of potential monetary benefits from using FMD viruses as vaccine strains that arise from comparison with the human health market. The profit generated from selling FMD vaccines is low compared to human vaccines and even as compared to other animal health products. These expectations can stifle the business case for developing new FMD vaccines, and consequently companies may redirect investment to lower risk activities and faster growing markets (26). This has a negative impact on global vaccine security with overall fewer FMD vaccines being produced and the absence of investment into updated high-quality and antigenically relevant vaccines. Similar constraints exist for other commercial actors such as diagnostic companies and pharmaceutical companies that may wish to utilise FMDV materials for new tests or therapeutics (Figure 2). The observation that there have not been any major epizootics to date involving countries blocking access to strains of FMDV for which current vaccines are ineffective does not diminish the need to be prepared for when such a situation arises.

### 2.3 Research projects and researchers

Beyond the utilisation of materials by commercial companies, the Nagoya Protocol presents a highly complex framework for scientists who work in the academic sector (including universities, governmental institutes, and not-for-profit organisations). Access to viruses, samples and data is often central to efforts to facilitate the development of prototype diagnostics, therapeutics, and vaccines and is also widely exploited to establish novel tools to understand the evolution, mechanisms of replication and infection, pathogenesis, immune responses, and host-cell interactions of FMDV. This work typically involves transnational collaborations with researchers in countries from which the materials have been sourced. Significant time and valuable resources in these institutions may be required to agree ABS terms associated with projects that might yield limited benefits. In addition, researchers in provider countries may be disadvantaged through reduced collaboration opportunities, whether through reluctance from external scientists, or as a result of their own lack of motivation stemming from insufficient advice, information, and assistance when negotiating ABS exchanges (17).

### 2.4 Provider countries

A perhaps overlooked group of stakeholders that is also impacted by restrictions on FMD research and development are farmers and livestock keepers in the provider countries. Tools and products arising from the utilisation of genetic resources can directly improve livestock productivity in those countries that have provided the material (Figure 2). For example, most countries in Africa and Asia do not currently have the capacity to manufacture FMD vaccines at sufficient volumes and quality required to have long-term impacts on disease control. Therefore, many endemic countries rely on FMD vaccines from external international companies, which are often located in the Global North. Even where suitable local manufacturing facilities exist, there is frequently a need for international cooperation and exchange of potential seed viruses, particularly when developing vaccines based on newly emerged strains of FMDV. There is a danger that the constraints brought about through the Nagoya Protocol will result in reduced access to new vaccines that are suited to the epidemiological situation. Even with the ABS legislation in the hands of the relevant country’s government, the complexities in achieving PIC and MAT can severely impede and protract the resource sharing process, leading to unfavourable timelines to develop new tools. These problems are often compounded by a lack of communication between the Nagoya Protocol NFPs and those government officials that direct FMD control and understand the urgency and benefits of the products that arise from the utilisation of materials. This can end up being detrimental not only to provider countries themselves but also to other countries in the region which are linked through circulation of epidemiologically related strains for FMD.

## 3 Potential measures to reduce unwanted impacts of the Nagoya Protocol

The potential problems described above create legal uncertainty for both users and providers of FMDV genetic material and DSI. Consequently, it is important to identify possible solutions to reduce the legal uncertainty. To facilitate sharing of materials and rapid availability of tools to control WOAH listed diseases, Resolution 15 of the 81st General Assembly of the WOAH (formerly OIE) in 2013 called on OIE Member Countries to report outbreaks of FMD to the OIE and to share FMD viral material and information about FMD viruses with OIE Reference Laboratories. Article 8(b) of the Nagoya Protocol explicitly calls upon states to ensure that the normal ABS rules and procedures do not interfere with public health emergencies or, as detailed in 8(c), with food and agriculture (and consequently food security). However, only a small number of countries have translated these articles into their national law, and even fewer have implemented measures to fast-track pathogen sharing in the face of an imminent emergency.

The CBD secretariat with contributions from various United Nations structures oversee the operation of the CBD and the Nagoya Protocol. To achieve an over-arching solution at international level that includes FMD, these groups, together with the Convention of the Parties to the Protocol, would first need to agree that a high level solution is actually required to address the challenges identified in this paper and then that the solution identified either operates within the context of the Nagoya Protocol or operates in a manner consistent with the principles of the Nagoya Protocol, as is the case for Specialised International Instruments referred to in Article 4.4 of the Nagoya Protocol. The Kunming-Montreal Global Biodiversity Framework may help to steer such a solution with its more holistic approach and target to foster joint technology development and strengthen scientific research and monitoring capacities.

Solutions have been identified for some human diseases. WHO has played a prominent role to facilitate the negotiation of a solution between the interested parties and, in cases such as the Pandemic Influenza Preparedness (PIP) framework, plays a functional role in acting as the repository and agreement holder for transfer of genetic material. Under the auspices of the WHO an Intergovernmental Negotiating Body is currently negotiating a WHO Convention, agreement or other international instrument on pandemic prevention, preparedness and response. This is being referred to as the Pandemic Accord. Article 12 of the most recent draft (May 22, 2023) of the Pandemic Accord contains the WHO Pathogen Access and Benefit Sharing System (the PABS System) for pathogens with pandemic potential in humans. There is no single institution that plays the same role as the WHO in the veterinary domain. International collaboration for veterinary diseases is managed by cooperation between the WOAH and the FAO, each respecting their particular mandate and responsibilities. WHO may also be involved, particularly when there is a zoonotic perspective to the health issue concerned (e.g., rabies and antimicrobial resistance). In the case of FMD, both WOAH and FAO are involved in efforts to control and eradicate the disease, particularly through the Global Framework for the Progressive Control of Transboundary Animal Diseases, except for the Americas, where the Pan American Health Organization, the Regional Office for the Americas of the WHO, has been coordinating the FMD eradication efforts in this region through its Pan American Foot and Mouth Disease Centre (PANAFTOSA), Rio de Janeiro, Brazil.

Key to any solution for FMD is building trust between providers and recipients, increasing awareness of the very substantive socio-economic benefits provided by effective FMD vaccines, and gaining a broader understanding of what benefits, monetary or otherwise, are both feasible and have a positive impact on animal health, One Health and biodiversity, taking into consideration all objectives of the CBD. In this context, benefits may extend beyond vaccines to support for capacity building and surveillance, technology transfer, and promoting innovation and scientific cooperation.

The objective of this paper is to raise awareness of the issues that exist in relation to FMD, to place these issues in the context of the experience gained from other diseases, and to highlight a number of potential elements that could be included in any solution for FMD. It is beyond the scope of this article to go into detail on the benefits and drawbacks of the various options or to explore their feasibility or desirability in detail. In developing any solution for FMD, it is of key importance to establish a system that ensures access and exchange of FMD strains whilst safeguarding the effective management of ABS issues related to FMD. Taking these primary objectives into account, the following possible elements might be considered in developing an overall solution. These elements are not mutually exclusive and could be implemented progressively in order to address the issues identified both pragmatically in the short to medium term and more strategically in the longer term.

1. The possibility of exempting FMDV from the scope of the Nagoya Protocol is superficially attractive but is not considered an appropriate or viable approach as it would be in direct conflict with the policy objectives of the CBD and is therefore not considered further.
2. **Raising awareness and consideration** of the Nagoya Protocol and related ABS frameworks amongst all stakeholders is considered an essential first step in developing further any potential solutions. These initiatives should be focussed on the perspective of the provider countries to ensure that all parties understand and respect the principles of the CBD. Furthermore, efforts to facilitate improved lines of communication between government officials in countries from which materials are sourced would also be beneficial (such as the Nagoya NFPs, NCAs and the Chief Veterinary Officers who typically direct national disease control initiatives). Lack of local awareness of the importance of FMD as a disease that impacts the livelihood of livestock producers and food security may arise since the disease exclusively affects livestock and may be considered of lower priority to the regulatory agencies involved in negotiating MAT than human pathogens.
3. Inclusion of standardised terms within the **Terms of Reference** for WOAH/FAO FMD reference laboratories would be useful to cover the sharing of FMD viruses between laboratories within the network and, separately, to clarify and standardize the approach to be followed to comply with national ABS requirements when viruses are shared between network laboratories and third parties for utilisation for other purposes. This network-wide standardisation of the approach to addressing ABS arrangements could take account of the experience gained from the routine exchange of seasonal influenza viruses between members of the GISRS, and the exchanges between the GISRS and the human pharmaceutical industry that follow recommendations for changes in vaccines strains by the WHO.
4. Formulation of a **specialised multilateral ABS instrument**, such as the WHO PIP or the International Treaty on Plant Genetic Resources for Food and Agriculture, that would have FMD within its scope may be a feasible goal in the longer term. Such a specialised international ABS instrument could reduce transaction costs and provide for more legal certainty for all stakeholders. The scope of such a solution would depend on the ambition and level of support that it could attract. Scopes such as pathogens causing epizootic diseases of animals, all veterinary pathogens or even pathogens of plants, animals and humans are all possible and would need to be considered in the initial phase of developing any overarching instrument.
5. Establish an international FMDV repository (“bank”) for the benefits of the international community, under agreements with the FAO, with the option to expand to other veterinary viruses of importance at a later stage. The experience gained in establishing the **European Virus Archive** (EVA: https://www.european-virus-archive.com/) and the WHO BioHub System for Preparedness and Response to Epidemics and Pandemics should be examined for applicability to FMD. The intention should be to simplify access to viruses in the repository for utilisation, including for use as a vaccine strain, by ensuring ABS compliance in advance so that access to samples could be achieved using a standard material transfer agreement.

Whatever solution is ultimately chosen will require political support from the wide range of stakeholders involved, together with a source of funding to cover the administrative and logistical costs that are inevitably required to assure exchange of materials in compliance with the Nagoya Protocol. Focus in the short term could be directed to simple solutions to facilitate exchange of FMD materials between willing partners on a voluntary basis whilst longer term solutions, possibly with wider scope than FMD alone, are sought at international level.

## 4 Conclusion

The application and potential negative impacts of the Nagoya Protocol and related ABS frameworks to viral pathogens is a topic of ongoing debate. The unintended negative impacts of the implementation of the Nagoya Protocol and the concept of viral sovereignty on pathogen research and development, or in outbreak situations, are well-documented with real life examples for viruses including SARS-CoV-2, Zika virus, and Middle East Respiratory Syndrome coronavirus (5, 15, 20, 22, 27–30). Similar debates have also occurred for other microbial pathogens (31, 32).

The purpose of this paper is to highlight the consequences for animal health that arise with respect to FMD as a result of the current ways in which the Nagoya Protocol is being implemented. Taken together, it seems prudent that all actors involved in the collection, testing and utilisation of FMD materials work on a common approach that delivers both fair and equitable sharing of benefits in line with the Nagoya Protocol and enables the continued rapid sharing of FMDV. In the absence of a solution to these challenges, the long-term consequences could be extremely detrimental for national and international initiatives to control FMD, including reduced availability of vaccines and diagnostic kits, breakdown of international partnerships, and withdrawal of pharmaceutical companies from this sector.

## Author contributions

JHo: Conceptualization, Writing – original draft, Writing – review & editing. EA: Conceptualization, Writing – original draft, Writing – review & editing. LK: Writing – review & editing. KB: Writing – review & editing. AC: Writing – review & editing. EC: Writing – review & editing. PE: Writing – review & editing. SG-N: Writing – review & editing. DG: Writing – review & editing. LG: Writing – review & editing, Conceptualization, Writing – original draft. SG: Writing – review & editing. LH: Writing – review & editing. PH: Writing – review & editing, Conceptualization, Writing – original draft. JHy: Writing – review & editing. MI: Writing – review & editing, Conceptualization, Writing – original draft. AK: Writing – review & editing. DL: Writing – review & editing. DM: Writing – review & editing, Conceptualization, Writing – original draft. SM: Writing – review & editing. FM: Writing – review & editing. CN: Writing – review & editing. M-KP: Writing – review & editing. EP: Writing – review & editing. FR: Writing – review & editing. FS: Writing – review & editing. HU: Writing – review & editing. PV: Writing – review & editing. WV: Writing – review & editing. DK: Writing – review & editing, Conceptualization, Resources, Writing – original draft.

## Footnotes

^1^ www.foot-and-mouth.org
